# Outcome Prediction by 40-Hz Steady-State Response After Large Hemispheric Infarction

**DOI:** 10.3389/fneur.2018.01093

**Published:** 2018-12-17

**Authors:** Yao Wang, Kaibin Huang, Shengnan Wang, Honghao Wang, Zhong Ji, Suyue Pan, Yongming Wu

**Affiliations:** Department of Neurology, Nanfang Hospital, Southern Medical University, Guangzhou, China

**Keywords:** 40-Hz steady-state response, large hemispheric infarction, neurocritical care, mortality, poor prognosis

## Abstract

**Background and Purpose:** The 40-Hz steady state response (SSR) reflects early sensory processing and has the potential to differentiate disease severity. This study aims to evaluate the predictive value of 40-Hz SSRs on the prognosis of patients with large hemispheric infarction (LHI).

**Methods:** We conducted a retrospective study in patients with LHI admitted to the neurological intensive care unit (NICU) of Nanfang Hospital, Southern Medical University, Guangzhou, China, between June 2008 and December 2014. Forty-hertz SSRs were recorded within 72 h of onset and categorized into 3 grades. The correlation between 40-Hz SSR grading and clinical outcome was examined.

**Results:** Of the 97 eligible participants, 41 (42.3%) died within 30 days and 68 (70.1%) exhibited a poor outcome (modified Rankin Scale of 5 and 6) at 90 days after the onset of LHI. We found that 40-Hz SSRs correlated significantly with NIHSS scores at admission and patient outcome. Moreover, Grade III 40-Hz SSR (bilateral sine waves that either disappeared or were not clearly identifiable) had a specificity of 97% and a positive predictive value of 94% in predicting 90-days poor outcome; Grade III 40-Hz SSR also had a specificity of 91% and a positive predictive value of 74% in predicting 30-days mortality.

**Conclusions:** 40-Hz SSR could be used as a simple and specific method in predicting poor prognosis after LHI.

## Introduction

Large hemispheric infarction (LHI) have the potential to cause severe cerebral edemas and cerebral herniations, leading to death and morbidity even when treated as conservatively as possible (1). Several methods have been developed to identify LHI patients at risk of progressing to a malignant stage (2, 3), given the growing body of evidence that suggests timely treatment with decompressive craniectomy may improve clinical outcomes (4). However, decompressive craniecto1my is not suitable for all patients and, although the procedure reduces mortality, it may also increase the number of severely impaired individuals (5, 6). The ability to reliably predict which LHI patients may develop an undesirable prognosis is an area of research that is significantly lacking. At present, the infarct volume under neuroimaging maintains a pivotal role for the estimation of prognosis, with robust associations of clinical relevance (7, 8). However, serial imaging might be difficult to perform in critically ill patients. The use of additional outcome parameters that could simply and repeatedly be obtained and reflect different aspects of stroke damage may be able to help specify prognoses in the early phases of LHI.

Neuronal networks in the sensory cortices, comprised of sensory cortical neurons, have a unique capability of entraining faithfully to the driving stimuli when subjected to phasic inputs. This entrainment to rhythmic sound stimuli is often referred to as the auditory steady-state response (SSR) (9, 10). A maximum SSR to a driving frequency of approximately 40 Hz was first reported by Galambos et al. (11). The 40-Hz SSR was used to test hearing integrity in infants as well as in audiometric tests for people of all ages (12). It has been suggested as an index for monitoring anesthetic depth because of its sensitivity to most general anesthetics (13, 14), with the exception of ketamine (14–17). Changes in amplitude or in latencies of the response would be observed with the effects of lesions (18–21). Since a 40-Hz SSR represents simultaneous activation over widely spaced areas of the brain, the change in its amplitude or latencies can reflect dysfunction in the brain and thus, the 40-Hz frequency may be valuable in the evaluation of neurological function (13, 22, 23). In a study of comatose patients after severe head injury or intracerebral hemorrhage, the presence or absence of a 40-Hz SSR correlated closely with clinical course and outcome (24). However, few studies have addressed the application of 40-Hz SSR in patients with LHI.

The present study was designed to determine the predictive value of 40-Hz SSRs in clinical outcomes for LHI patients under the most conservative treatments.

## Methods

### Study Design and Participants

The retrospective study was conducted in patients with LHI (8) admitted to the neurological intensive care unit (NICU) of Nanfang Hospital, Southern Medical University, Guangzhou, China, between June 2008 and December 2014. The inclusion criteria were: (1) a diagnosis of acute ischemic stroke affecting the total or subtotal territory of the middle cerebral artery (MCA), at least partially involving the basal ganglia and with or without involvement of the adjacent (i.e., anterior cerebral artery or posterior cerebral artery) territories, as confirmed by neuroimaging (8); (2) aged >18 years; and (3) a 40-Hz SSR test having been completed within 72 h of onset. Exclusion criteria included: (1) a pre-stroke score of ≥1 on the modified Rankin scale (mRS) or of < 95 on the Barthel index; (2) suffering from end-stage malignant diseases that might confound the prognosis; (3) known diseases of hearing or peripheral nerves; (4) antiepileptic or sedative medications having been administered before the 40-Hz SSR assessment; and (5) endovascular treatment having been conducted during the study period.

According to national and international guidelines for the management of acute ischemic stroke (8), all participants received a maximum conservative treatment or a decompressive craniectomy, with the latter being performed in cases of increased intracranial pressure and herniation, and codetermined by the neurosurgeon and legal representatives independent of the study design.

### 40-Hz SSR Tests and Grading

40-Hz SSR tests were recorded within 72 h after the onset of LHI, according to standard techniques established for using the Viking Quest system evoked potential equipment (Nicolet Company, American). Briefly, silver-chloride electrodes were applied to the scalp at C_Z_, A1, and A2 according to the international 10–20 system of electrode placement, and a ground electrode was placed on the forehead, at the Fpz position. To record 40-Hz SSR, repeated pips at a rate of 39.1 Hz were generated in standard inserted headphones. Monaural stimulation was used with 95 decibels (dB) nHL tone pips, and the ear contralateral to stimulation was masked with 70 dB white noise. We determined the interpeak amplitude between the first positive peak (P1) and the first negative wave (N1) as N1 amplitude with a 100 ms time base (11). Internal laboratory standards were established in an independent group of young healthy volunteers (*n* = 80) (Supplementary Method, Supplementary Figure 1).

**Figure 1:**
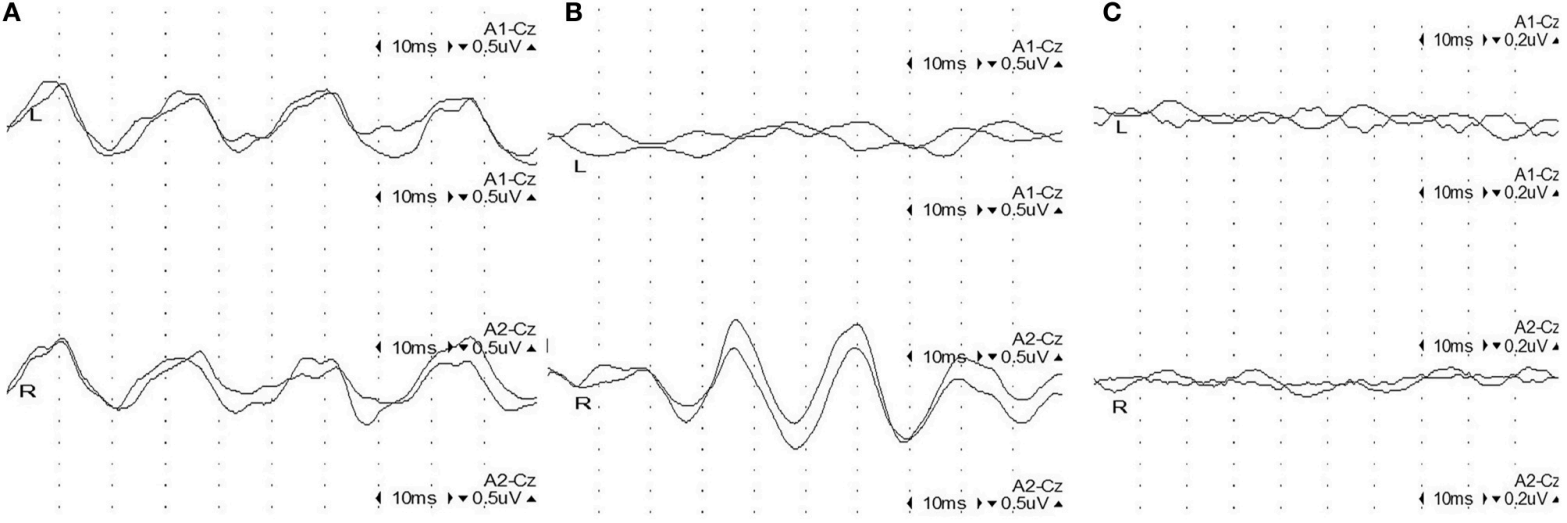
Representative figures of 40-Hz steady-state responses (SSRs) in 3 grades. **(A)** Grade I: normal 40-Hz SSR: 4 sine waves appearing regularly in 100 ms with settled intervals, with a comparable amplitude on both sides. **(B)** Grade II: unilateral abnormal 40-Hz SSR: sine waves on the left side are extinct, while 4 sine waves appear regularly on the right side in 100 ms with settled intervals and normal amplitude. **(C)** Grade III: bilateral abnormal 40-Hz SSR: sine waves are extinct bilaterally.

Amplitudes of the 40-Hz SSR were considered abnormal when the side-to-side difference exceeded 50% when compared to the unaffected contralateral response or when the amplitude was below the 2.5-fold value of the established standard value (Supplementary Table 1). Accordingly, 40-Hz SSRs were classified as one of 3 grades (25) (Figure 1): Grade I (normal amplitude), with bilateral sine waves appearing clearly; Grade II (moderate abnormal amplitude), with unilateral sine waves not present, or an amplitude ratio lower than 50% of the unaffected side; and Grade III (severe abnormal amplitude), with bilateral sine waves not present or not clearly identifiable.

### Clinical Data

Demographic, clinical, and neuroimaging data were collected from our prospectively organized database by a research associate (S.W.) and two neurologists (H.W. and Z.J.), all of whom were blind to the 40-Hz SSR data. All participants were followed up for 90 days after the onset of LHI by a trained neurologist blind to the study data via telephone interviews. The primary outcome was a functional prognosis at 90 days, which was classified as good (mRS of 0 to 4) and poor (mRS of 5 and 6) (26). The secondary outcome was mortality of all causes at 30 days.

### Statistical Analysis

Continuous data were presented as the mean ± standard deviation (SD) or median (25–75% interquartile range [IQR]) and compared by Student's *t*-test or Mann-Whiney *U* test, as appropriate. Differences in proportion among categorical data were assessed using chi-squared test or Fisher's exact test. Correlations between the variables were determined with the Spearman correlation test. We calculated the sensitivity, specificity, positive predictive value (PPV), and negative predictive value (NPV) of predefined thresholds with 40 Hz SSR Grade III to predict 90-days functional outcomes and 30-days mortalities, and reported the corresponding 95% confident intervals (CIs) (27). The level of significance was set to *p* < 0.05. All statistical analyses were performed with SPSS 20.0 software (SPSS Inc., Chicago, IL, USA).

## Results

**Figure 2:**
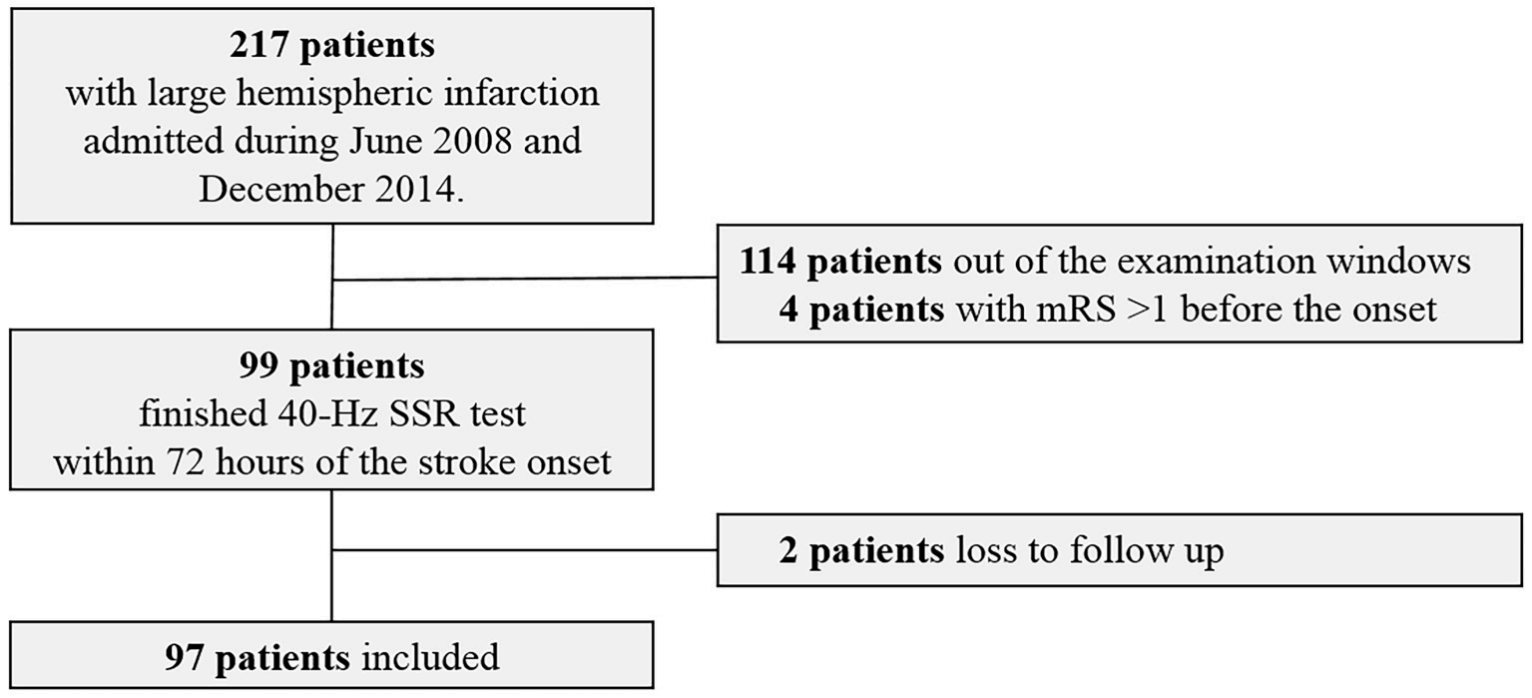
Patient inclusion flowchart.

Between June 2008 and December 2014, 97 eligible LHI patients were recruited (Figure 2). Among them, 41 (42.3%) patients died within 30 days after the onset of LHI (Supplemental Table 2) and 68 (70.1%) patients had a poor outcome at 90 days (Table 1). The results showed that patients with poor outcomes had higher NIHSS scores at admission as compared to those with good outcomes. For the causes of the strokes, more patients in the poor outcome group could be attributed to cardioembolisms. However, beyond that, we did not observe significant difference between the good and poor outcome groups in age, gender, the ratio receiving intravenous thrombolysis or decompressive craniectomy, medical history or laboratory findings at admission. Similar results were observed between survivors and nonsurvivors at 30 days (Supplementary Table 2).

**Table 1:** Baseline characteristics between patients with good (mRS 0–4) and poor (mRS 5–6) outcome at 90 days.

**Parameters**	**Good outcome (*n* = 29)**	**Poor outcome (*n* = 68)**	***P-*value**
Age [y], median (IQR)	67 (53, 76)	68 (57, 76)	0.611
Male, *n* (%)	18 (62.1)	41 (60.3)	0.870
NIHSS score on admission, mean ± *SD*	15.7 ± 6.0	20.5 ± 7.8	0.004
Intravenous thrombolysis, *n* (%)	4 (13.8)	8 (11.8)	0.953
Decompressive craniectomy, n (%)	1 (3.4)	7 (10.3)	0.472
**Medical history**, ***n*** **(%)**			
Hypertension	18 (62.1)	43 (63.2)	0.913
Diabetes mellitus	5 (17.2)	15 (22.1)	0.591
Atrial fibrillation	5 (17.2)	17 (25.0)	0.404
Myocardial infarction	1 (3.4)	2 (2.9)	1.000
Temperature [°C], median (IQR)	36.6 (36.5, 37.3)	36.9 (36.6, 37.6)	0.078
Systolic blood pressure [mmHg], mean ± *SD*	152.1 ± 26.1	144.6 ± 30.8	0.262
Stenosis or occlusion of the ipsilateral extracranial ICA, *n* (%)	0 (0)	4 (44.4)	1.000
Stenosis or occlusion of the contralateral extracranial ICA, *n* (%)	7 (25.9)	24 (36.4)	0.332
**Vessel occlusion dichotomized**, ***n*** **(%)**			0.205
Isolated MCA	21 (72.4)	40 (58.8)	
ICA + MCA	8 (27.6)	28 (41.2)	
**Etiology (TOAST classification)**, ***n*** **(%)**			0.047
Large artery atherosclerosis	23 (79.3)	35 (51.5)	
Cardioembolism	6 (20.7)	27 (39.7)	
Small vessel disease	0 (0)	0 (0)	
Other determined etiology	0 (0)	5 (7.3)	
Undetermined origin	0 (0)	1 (1.5)	
**Laboratory values**			
Hematocrit [%], mean ± *SD*	39.5 ± 7.3	39.1 ± 7.1	0.829
White blood cell count [ × 10^6^/mL], mean ± SD	10.9 ± 3.8	12.7 ± 4.8	0.075
Creatinine [μmol/L], median (IQR)	76 (59, 104)	80 (63, 105)	0.512
40 Hz SSR, median (IQR)	1 (1, 2)	1 (1, 2)	0.046
40 Hz SSR grade III, *n* (%)	1 (3.4)	15 (22.1)	0.034

**Figure 3:**
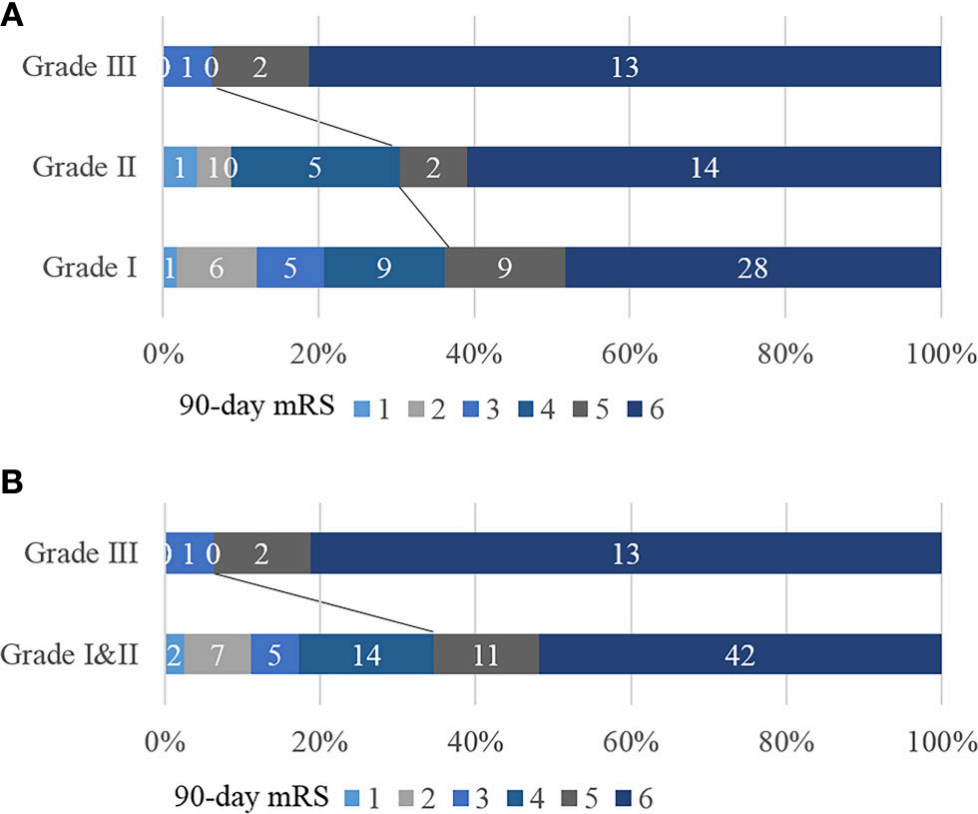
Correlation between 40-Hz SSR grading and mRS scores. **(A)** 40-Hz SSRs were categorized into Grade I, II, and III. **(B)** 40-Hz SSRs were categorized into Grade I & II vs. Grade III.

In terms of the 40-Hz SSR testing results, the median grade in the poor outcome group was higher than that in the good outcome group. Additionally, more patients in the poor outcome group received a Grade III classification than those in the good outcome group (Table 1). The correlation between 40-Hz SSR grades and 90-days prognoses remained when the functional outcome was treated as specific mRS scales (Figure 3A, Table 2). Correlation analysis also revealed a close correlation between NIHSS scores and 40-Hz SSR grades, indicating that the 40-Hz SSR could accurately reflect the severity of the LHI (Table 2).

**Table 2:** Correlation between 40-Hz SSRs (Grade I & II vs. Grade III) and NIHSS, 90-days mRS and duration of hospital stay.

**Parameters**	***R***	***P*-value**
NIHSS score on admission	0.244	0.016
90-days mRS	0.232	0.022
Duration of hospital stay	−0.181	0.108

To further evaluate the performance of 40-Hz SSRs in predicting patient outcomes, we categorized the 40-Hz SSRs into Grade I & II vs. Grade III (Figure 3B). It was found that 40-Hz SSRs that were Grade III had a sensitivity of 22%, specificity of 97%, PPV of 94%, and NPV of 35% in predicting 90-days poor outcomes (Table 3). Additionally, 40-Hz SSRs that were Grade III had a sensitivity of 34%, specificity of 91%, PPV of 74%, and NPV of 65% in predicting 30-days mortalities (Table 3).

**Table 3:** The performance of 40-Hz SSR Grade III patients in prediction of 90-days poor outcome and 30-days mortality.

**Outcome**	**Sensitivity**	**Specificity**	**PPV**	**NPV**
90-days poor outcome	0.22 (0.13–0.34)	0.97 (0.80–1.00)	0.94 (0.68–1.00)	0.35 (0.25–0.46)
30-days mortality	0.34 (0.21–0.51)	0.91 (0.80–0.96)	0.74 (0.49–0.90)	0.65 (0.54–0.76)

*PPV, positive predictive value; NPV, negative predictive value*.

## Discussion

The present study demonstrated the 40-Hz SSR as an outcome prediction tool for patients with LHI. The results showed that 40-Hz SSRs of Grade III had a high specificity and PPV in predicting both 90-days poor prognosis and 30-days mortality after LHI under the maximum conservative treatments, indicating that the 40-Hz SSR could be reliably used to assess a poor outcome after LHI.

Early prediction of clinical outcomes after an ischemic stroke is essential for the planning of acute and rehabilitative therapeutic strategies during the first days of hospital care. Early changes seen in cranial CT, MRI and angiography are all valuable for predicting malignant edemas after LHI (8). For instance, the presence of carotid T occlusions on angiographies predicted fatal outcomes with a positive predictive value of 47%, a negative predictive value of 85%, a sensitivity of 53%, and a specificity of 83% (28). However, neuroimaging might be difficult to complete for critically ill patients, especially when dynamic evaluation is required. Neuroelectrophysiology is an important part of neurological assessment in patients treated in the NICU, with great advantages in both bedside and dynamic evaluation. Current practice guidelines have suggested both brainstem auditory evoked potentials (BAEP) and EEG as complimentary methods to predict a malignant course within the first 24 h after LHI (8). Pathological response to BAEP exhibits a specificity of 79% and a PPV of 79% in the prediction of a malignant course in patients suffering from severe ischemic MCA syndromes (25). In stroke patients with initial paralysis of the upper extremities, the presence or absence of motor evoked potentials have a similar predictive value compared to early clinical assessment in regards to long-term motor recovery in the hands (29). Our study adds to the current knowledge that 40-Hz SSRs could be used to predict a fatal prognosis after LHI, with high specificity and PPV.

The 40-Hz SSR was proposed because an input frequency of approximately 40-Hz taps into the natural resonance frequency of auditory cortical neural assemblies, leading to a larger recruitment and a greater response in electroencephalograms (9, 30). The 40-Hz SSR represents a robust entrainment of auditory, cortical, and other networks involved in the auditory processing of sound that respond particularly well to click or tone stimuli presented in the gamma frequency. As previously reported, the 40-Hz SSR has, as its important source, the primary and secondary auditory cortices, while brainstem and forebrain structures also contribute to the scalp generated signal (22, 23, 31, 32). Therefore, if the occlusion of the MCA main stem impairs these structures, the 40-Hz SSR will represent a deficit response. Previous research has indicated that acute N-methyl-d-aspartate receptor seems to play an important role in the regulation of the 40-Hz SSR (33). In patients with Alzheimer's disease, the 40-Hz SSR power is significantly increased compared to mild cognitive impairment subjects and healthy controls, indicating that the 40-Hz SSR can reliably be used to measure disease progression (34).

Both clinical trials and animal models have demonstrated the high sensitivity of 40-Hz SSRs for consciousness level monitoring. As the severity of the disease progresses, the 40-Hz SSR amplitude decreases or disappears, and the results of 40-Hz SSRs are easier to read compared to traditional evoking potentials such as BAEPs. Neurologists simply need to recognize whether the 4 sine waves appear regularly and whether the amplitude ratio is lower than 50% of the referential range.

This study has several limitations. First, although all participants had completed 40-Hz SSR evaluation within 72 h, the exact time point differed among subjects. Thus, a further study with 40-Hz SSRs performed earlier after the stroke onset, possibly within 24 h, will be required to confirm the present findings. Second, this study was initially designed to evaluate the predictive value of 40-Hz SSRs in LHI patients with conservative treatment, before current evidence that supports the use of endovascular treatment in acute ischemic strokes. Therefore, another study utilizing a group of patients under endovascular treatment should be completed. Third, as a retrospective study, the potential selection and information bias in this study could not be completely avoided.

## Conclusion

Our study initially indicates that the 40-Hz SSR can be used as a simple, reliable and specific tool in predicting early death and unfavorable prognosis in LHI under maximum conservative treatments.

## Ethics Statement

This study was approved by the Nanfang Hospital's ethics committee on clinical research. Informed consent was waived by the review board because of the pure observational and retrospective nature of the study.

## Author Contributions

YoW contributed to study conception and design. YaW performed the 40-Hz SSR tests and drafted the manuscript. KH performed statistical analysis and helped to revise the manuscript. SW collected clinical data. HW and ZJ reviewed neuroimaging data and participated in patient follow up. SP participated in study conception and helped to revise the manuscript. All authors made substantial contributions. All authors read and approved the final version of the manuscript.

### Conflict of Interest Statement

The authors declare that the research was conducted in the absence of any commercial or financial relationships that could be construed as a potential conflict of interest.
