# Supplementary material for: Outcome Prediction by 40-Hz Steady-State Response After Large Hemispheric Infarction
Source: Front Neurol. 2018 Dec 17;9:1093. doi: 10.3389/fneur.2018.01093 (PMC6304417; doi:10.3389/fneur.2018.01093)
Supplement: Supplementary file 1 [file Data_Sheet_1.docx]

**Supplementary Information**

**Supplementary Method**

**Healthy Volunteers Recruitment**

To determine the referential amplitude under normal physiological conditions, 80 healthy volunteers aged > 18 years were recruited to receive 40-Hz steady-state response (SSR) test. Exclusion criteria were: (1) with a pre-stroke score of ≥ 1 on the modified Rankin scale (mRS) or of < 95 on the Barthel index; (2) known diseases of hearing or peripheral nerves.

**Measurement of 40-Hz SSR Amplitude**

40-Hz SSR tests were performed as described in the Method section of the manuscript. We determined the interpeak amplitude between the first positive peak (P1) and the first negative wave (N1) as the amplitude of N1 with a 100 msec time base ^1^ (**Supplementary Figure I**). In the same manner, the amplitude of N2, N3, and N4 were identified. The demographic data and average amplitude of N1, N2, N3, and N4 in bilateral sides were shown in **Supplementary Table 1**.

**
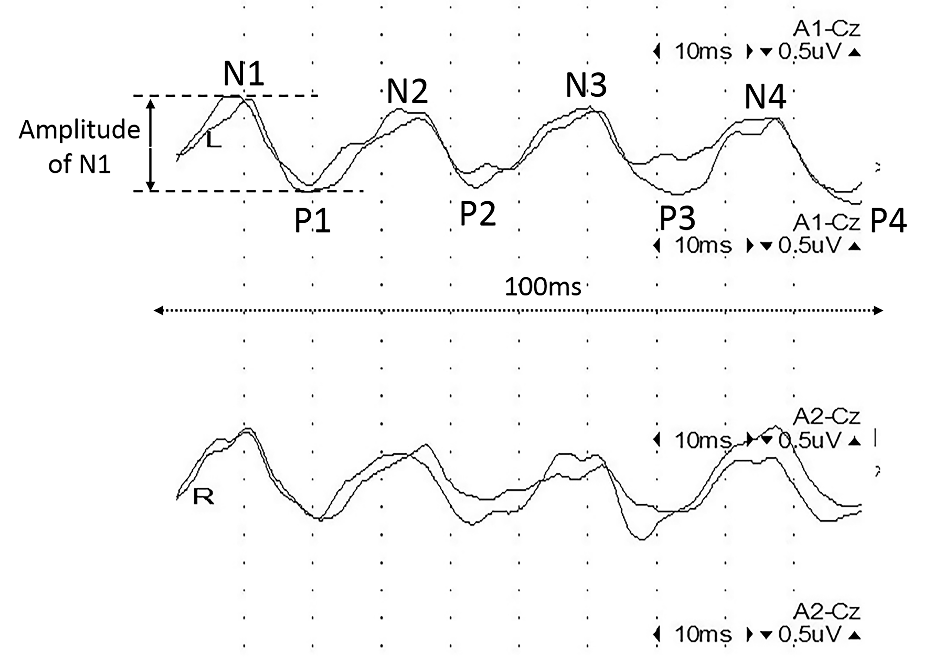
**

**Supplementary Figure I.** The amplitude of N1 was determined by the interpeak amplitude between the first positive peak (P1) and the first negative wave (N1).

**Supplementary Table I.** Demographic date and 40-Hz SSR amplitudes in 80 healthy volunteers.

| Parameters | Value |
| --- | --- |
| Age [years], mean ± SD | 42 ± 13 |
| Male (n, %) | 44 (55%) |
| 40-Hz SSR amplitude [μV], mean ± SD |  |
| N1 (L) | 1.57 ± 0.83 |
| N2 (L) | 1.66 ± 0.69 |
| N3 (L) | 1.54 ± 0.83 |
| N4 (L) | 1.58 ± 0.80 |
| N1 (R) | 1.66 ± 0.81 |
| N2 (R) | 1.65 ± 0.84 |
| N3 (R) | 1.62 ± 0.75 |
| N4 (R) | 1.59 ± 0.75 |

N, negative; L, left side; R, right side.

**Supplementary Table II.** Baseline characteristics between survivors and non-survivors at 30 days after the onset.

|  | Survivors  (*n* = 56) | Non-survivors  (*n* = 41) | *P*  value |
| --- | --- | --- | --- |
| Age [y], median (IQR) | 65.8 ± 13.7 | 64.1 ± 15.3 | 0.556 |
| Male, *n* (%) | 46 (60.7) | 25 (61.0) | 1.000 |
| NIHSS score on admission, , mean ± SD | 17.6 ± 7.0 | 21.1 ± 8.0 | 0.022 |
| Intravenous thrombolysis, *n* (%) | 8 (14.3) | 4 (9.8) | 0.503 |
| Decompressive craniectomy, *n* (%) | 3 (5.4) | 5 (12.2) | 0.403 |
| Medical History, *n* (%) |  |  |  |
| Hypertension | 37 (66.1) | 24 (58.5) | 0.525 |
| Diabetes mellitus | 11 (19.6) | 19 (22.0) | 0.804 |
| Atrial fibrillation | 9 (16.1) | 13 (31.7) | 0.069 |
| Myocardial infarction | 1 (1.8) | 2 (4.9) | 0.572 |
| Temperature [°C], median (IQR) | 36.9 ± 0.6 | 37.2 ± 0.9 | 0.062 |
| Systolic blood pressure [mmHg] , mean ± SD | 148.6 ± 25.8 | 144.6 ± 34.1 | 0.511 |
| Stenosis or occlusion of the ipsilateral extracranial ICA, *n* (%) | 1 (1.8) | 3 (7.3) | 0.423 |
| Stenosis or occlusion of the contralateral extracranial ICA, *n* (%) | 18 (32.1) | 13 (31.7) | 0.861 |
| Vessel occlusion dichotomized |  |  | 0.000 |
| Isolated MCA occlusion | 44 (78.6) | 17 (41.5) |  |
| ICA +MCA occlusion | 12 (21.4) | 22 (58.5) |  |
| Etiology (TOAST classification) |  |  | 0.053 |
| Large artery atherosclerosis | 39 (69.6) | 18 (43.9) |  |
| Cardioembolism | 15 (36.8) | 18 (43.9) |  |
| Small vessel disease | 0 | 0 |  |
| Other determined etiology | 1 (1.8) | 4 (9.8) |  |
| Undetermined origin | 1 (1.8) | 1 (2.4) |  |
| Laboratory values |  |  |  |
| Hematocrit (%), mean ± SD | 0.38 ± 0.07 | 0.41 ± 0.07 | 0.156 |
| White blood cell count [× 10^6^/ml], mean ± SD | 11.1 ± 4.1 | 13.5 ± 4.8 | 0.009 |
| Creatinine [μmol/L], median (IQR) | 84 (64, 109) | 79 (62, 103) | 0.498 |
| 40-Hz SSR, median (IQR) | 1 (1, 2) | 1 (1, 2) | 0.028 |
| 40-Hz Grade III | 5 | 14 | 0.000 |

**References**

1. Galambos R, Makeig S, Talmachoff PJ. A 40-Hz auditory potential recorded from the human scalp. *Proc Natl Acad Sci U S A*. 1981;78:2643-2647
